# Perceived Barriers to Fruit and Vegetable Gardens in Early Years Settings in England: Results from a Cross-Sectional Survey of Nurseries

**DOI:** 10.3390/nu11122925

**Published:** 2019-12-03

**Authors:** Sara E Benjamin-Neelon, Amelie A Hecht, Thomas Burgoine, Jean Adams

**Affiliations:** 1 Department of Health, Behavior and Society, 615 North Wolfe Street, Johns Hopkins University, Baltimore, MD 21205, USA; 2 UKCRC Centre for Diet and Activity Research (CEDAR), MRC Epidemiology Unit, University of Cambridge School of Clinical Medicine, Box 285, Institute of Metabolic Science, Cambridge Biomedical Campus, Cambridge CB2 0QQ, UK; tb464@medschl.cam.ac.uk (T.B.); jma79@medschl.cam.ac.uk (J.A.); 3 Department of Health Policy and Management, 624 North Broadway, Johns Hopkins University, Baltimore, MD 21205, USA; ahecht3@jhu.edu

**Keywords:** Child Care, Early Care and Education, Gardens, Produce

## Abstract

Garden-based interventions may increase child intake of fruits and vegetables and offset food costs, but few have been conducted in early care and education (ECE). This study assessed whether nurseries were interested in and perceived any barriers to growing fruits and vegetables. Surveys were mailed to a cross-sectional sample of nurseries in 2012–2013 throughout England. Nurseries were stratified based on socioeconomic status as most, middle, or least deprived areas. We fit logistic regression models to assess the odds of nurseries interested in growing fruits and vegetables and perceiving any barriers, by deprivation tertile. A total of 851 surveys were returned (54% response). Most nurseries (81%) were interested in growing fruits and vegetables. After adjustment, there was no difference in interest in the middle (OR 1.55; CI 0.84, 2.78; *p* = 0.16) or most (OR 1.05; CI 0.62, 1.78; *p* = 0.87) deprived areas, compared to the least deprived. Nurseries reported barriers to growing fruits and vegetables, including space (42%), expertise (26%), and time (16%). Those in the most deprived areas were more likely to report space as a barrier (OR 2.02; 95% CI 1.12, 3.66; *p* = 0.02). Nurseries in the most deprived areas may need creative solutions for growing fruits and vegetables in small spaces.

## 1. Introduction

Recent studies demonstrate that young children in high-income countries like the United States (US) and the United Kingdom (UK) consume insufficient servings of fruits and vegetables [1,2,3]. Children from low socioeconomic status families are particularly at risk for poor dietary intake [4,5,6], and interventions to increase fruit and vegetable consumption could be tailored to reach this vulnerable population [7]. Traditionally, the family has been the primary influence on young children’s dietary intake. Parent preferences and parents modeling healthy behaviors have been shown to be important determinants [8]. However, a growing number of parents share caregiving and feeding responsibilities with early care and education (ECE) providers [9,10], and these providers can impact children’s dietary intake [11]. In high-income countries, nearly 80% of children ages 3–6 years and 25% of children ages 0–3 years spend time in some form of non-parental child care [12]. Children may consume one-half to two-thirds of their daily calories in these settings [13], including most of their carbohydrates and servings of fruit [14]. 

There is ample evidence that young children consume inadequate amounts of fruits and vegetables in ECE. A previous study of children in the US found they consumed one-third of a serving of fruit and one-quarter of a serving of vegetables per day in ECE—far fewer than the recommended national guidelines [15]. Moreover, nearly 50% of vegetables consumed in ECE were fried potatoes, and only 8% were the more nutrient-dense dark green or brightly colored vegetables [15]. Other studies of young children in the US and the Netherlands have found similar results [14,16,17,18]. In the UK, limited data suggest that children are also served insufficient quantities of fruits and vegetables. In one study in Northern England, about half of nurseries (one type of ECE setting) reported serving either a fruit or vegetable with lunch daily [19]. In our prior work, we found that 92% of nurseries reported serving a fruit and 70% reported serving a vegetable to children each day [20]. Although somewhat promising, this still falls below the recommended amount [21]. 

Studies show that exposure to fruits and vegetables by age 5 years is vital to establishing habitual consumption later in life [22,23,24]. Encouraging children to try and accept novel foods has been more effective in younger children than with older children, and repeated exposure has yielded positive results in ECE setting [23,25,26,27,28]. Interventions that engage children in food preparation, encourage hands-on experiences, and include home-grown foods have increased child fruit and vegetable consumption beyond that of an intervention that merely increases availability [11,29,30,31,32,33,34,35]. Thus, interventions that include growing fruits and vegetables, for example, have the potential to increase child intake, but only a handful have been conducted in ECE [36]. The ECE setting is perhaps ideal because preschool-aged children may be more likely to try new foods like vegetables with both repeated exposure and eating in a group setting [25,27,28,37]. 

Further, gardens may help offset costs associated with the purchasing of fruits and vegetables. School gardens in high-income countries have focused on promoting experiential learning and exposing children to fruits and vegetables to increase familiarity and consumption [38,39,40]. An added benefit for ECE, however, is the potential to serve children what is grown. Generally, ECE programs tend to be smaller and there are often fewer children to feed, compared to schools. This may help offset food costs for fruits and vegetables served to children in ECE [41], in addition to other benefits.

The aims of this study were to assess whether nurseries were interested in growing their own fruits and vegetables and perceived any barriers to doing so. We further assessed whether responses to these questions differed by socioeconomic status. We hypothesized that nurseries in the most deprived areas of England would be more interested in growing their own fruits and vegetables as a means to offset food costs, but would report cost to establishing the garden as the primary barrier.

## 2. Materials and Methods

### 2.1. Study Design and Sample

For the cross-sectional Nutrition in Nurseries study, we mailed surveys to a stratified random sample of 2000 nurseries throughout England in 2012−2013. We obtained a list of registered nurseries in England from Ofsted, the organization responsible for regulating ECE programs in England. Ofsted defines nurseries to include any ECE setting that provides care for children on a regular basis that is not located on domestic premises. Thus, nurseries may include preschools or child care centers but not childminders or family child care homes (i.e., care on domestic premises). To stratify, we first used nursery addresses to geocode them at the postcode level, using geographic information system (GIS) (Arc GIS 10, ESRI Inc., Redlands, CA, USA) software. We used the geocoded data to categorize nurseries within lower super output areas (LSOA)—small administrative boundaries containing approximately 1,500 residents. Next, we stratified nurseries based on LSOA tertile of the index of multiple deprivation (IMD). IMD data were for 2010, which were the most recent scores available at the time of the survey. The IMD is published by the Department for Communities and Local Government in England and is a measure of income, employment, health and disability, education, barriers to housing, and crime. Expecting a lower response rate, we oversampled nurseries in the most deprived tertile. We mailed 1000 surveys to nurseries in the most deprived LSOAs, 500 to the middle and 500 to the least-deprived LSOAs. Additional information about the Nutrition in Nurseries study has been reported elsewhere [20].

### 2.2. Survey

A primary goal of the Nutrition in Nurseries study was to evaluate the nutritional quality of meals and snacks served in nurseries and assess consistency with mandatory and voluntary nutrition standards [20]. Secondarily, we assessed manager interest in growing fruits and vegetables at the nursery and perceived barriers to doing so in order to inform potential development and implementation of a future gardening intervention. We designed the survey to be completed by nursery managers in approximately 20 min. The survey assessed a number of factors related to nutrition and healthy food provision within the nursery. The final survey included 23 questions about food practices and the nutrition environment within the nursery; 16 demographic questions about children in the nursery and the manager; and two questions assessing the amount of time spent completing the survey. We used three existing surveys developed to assess nutrition and healthy eating practices in US-based ECE [42,43,44], modifying the questions for use in England. The first showed moderate to high validity and reliability [42]; the second demonstrated moderate to high reliability, but was not tested for validity [43]; and the third, to our knowledge, was not evaluated for either, though the authors note that it was based in part on the previous two surveys [44]. The survey was then reviewed by nutrition experts, parents of preschool-aged children, and nursery care providers in England prior to the launch of the study. We included a human subjects fact sheet with the mailed survey stating that completion constituted consent to participate in the study. Thus, all subjects gave their informed consent for inclusion before they participated in the study. The study was conducted in accordance with the Declaration of Helsinki, and the protocol was approved by the Ethics Committee of the University of Cambridge Psychology Research (Project identification code: Pre.2012.49).

We asked managers to report whether or not they were interested in growing their own fruits and vegetables at the nursery. If interested, we asked whether they were already growing fruits and vegetables at the nursery. We also asked whether managers perceived any barriers to establishing fruit and vegetable gardens. We then provided a list of potential barriers (e.g., time or expertise) developed by the research team and also allowed for write-in responses. We categorized these responses as cost, external threats (e.g., pests or vandalism would be a problem), ownership/shared use (e.g., the nursery did not have the authority to use the outdoor space for gardening), and seasonality or growing conditions (e.g., the nursery was closed during peak growing season or the soil was not suitable for growing fruits and vegetables). 

### 2.3. Analysis

We fit binary logistic regression models to assess the odds of interest in growing fruits and vegetables and perceiving any barriers to growing fruits and vegetables, by deprivation tertile with the least deprived tertile as the referent group. We adjusted for covariates that were of a priori interest based on prior studies [20,45], including nursery type (based in a workplace, run by a non-profit, or part of a corporate chain), total number of children enrolled, years in operation, and manager education (less than a 2-year degree or a 2-year degree or higher). Further, we included an indicator for urbanicity when we examined space as a barrier, as location in an urban versus rural area may explain any potential association. We present results in terms of odds ratios, 95% confidence intervals (CI), and two-sided *p*-values. We conducted all analyses using Stata version 14.1 (StataCorp LP, College Station, TX, USA) at a significance level of < 0.05.

## 3. Results

A total of 851 of nurseries returned a completed survey, resulting in a 54% response rate after accounting for surveys returned undelivered and nurseries that had closed for business, did not care for children regularly, or did not provide meals and snacks to children. The response rate was similar across all deprivation tertiles. Of the 851 surveys returned, 846 provided complete data for this analysis (five were missing information required to geocode nurseries). Nurseries had been in operation for a mean and standard deviation (SD) of 17.1 (12.3) years and over half (58%) of nursery managers had a 2-year degree or higher (Table 1). Nurseries were based in a workplace (43%), run by a non-profit (25%), or part of a corporate chain (33%). Nurseries had a mean (SD) of 49.9 (37.3) children enrolled (range 2−310); nearly all (97%) nursery managers were women.

Most nurseries (81%) were interested in growing their own fruits and vegetables (Table 2). Seventeen nurseries did not respond to the question and were therefore not included as not interested or interested in Table 2 below. Of those interested, 40% of nurseries were already growing some fruits or vegetables. Among all nurseries, a small percentage (18%) were not interested in growing fruits or vegetables.

In adjusted analyses, interest in growing fruits and vegetables did not differ by deprivation tertile (interest was not associated with deprivation in the middle (OR 1.55; 95% CI 0.84, 2.87; *p* = 0.16) or most deprived (OR 1.05; 95% CI 0.62, 1.78; *p* = 0.87) tertile, compared to nurseries in the least deprived tertile). Nursery managers reported a number of perceived barriers to growing fruits and vegetables, including space (42%), expertise (26%), and time (16%). However, 24% reported no barriers. Nurseries already growing, compared to those who were interested but not yet growing, were significantly more likely to report no barriers (OR 2.90; CI 1.87, 4.50; *p* ≤ 0.001) and significantly less likely to report space (OR 0.36; CI 0.24, 0.55; *p* ≤ 0.001), expertise (OR 0.30; CI 0.19, 0.49; *p* ≤ 0.001), or time (OR 0.58; CI 0.34, 1.0; *p* = 0.049) as a barrier. There were no significant differences between groups regarding likelihood of reporting cost, external threats, ownership/shared use, or seasonality/growing conditions as barriers. Of nurseries that were interested but not yet growing, those in the most deprived areas were more likely to report space as a barrier (OR 2.02; 95% CI 1.12, 3.66; *p* = 0.02) (Table 3). However, adjusting for urban location did not substantially change the results (OR 2.19; 95% CI 1.18, 4.06; *p* = 0.01). There were no other differences in perceived barriers by deprivation tertile.

## 4. Discussion

In this cross-sectional survey of nursery managers throughout England, we found that the majority were interested in growing their own fruits and vegetables and interest did not vary based on area deprivation level. This finding was contrary to our hypothesis. We expected nursery managers in the most deprived areas to be more interested in growing fruits and vegetables, in part to offset food costs. We also assessed barriers and hypothesized that costs associated with establishing gardens would be the primary barrier reported. Instead, very few nurseries reported cost as a barrier to growing fruits and vegetables and space was the most commonly reported barrier. 

Most nursery managers in our study expressed interest in growing fruits and vegetables. Gardens in ECE may expose children to fruits and vegetables through experiential learning, which may ultimately encourage greater consumption. In a recent qualitative study in the US, ECE providers reported that the ECE environment had the potential to exert a strong influence over children and was an ideal setting for introducing less familiar foods [46]. In another qualitative study in the Netherlands, managers believed it was vital to encourage healthy eating for the children in their care [47]. Managers emphasized the importance of making healthy foods, like fruits and vegetables, readily available in the ECE environment [47]. 

Moreover, child care providers in the US believed that fruit and vegetable gardens could help increase children’s willingness to try new foods [46]. A handful of prior gardening interventions have been conducted in ECE settings [48,49,50]. One observed a modest increase in vegetable but not fruit intake in children [48], another found the intervention itself to be well received but did not measure dietary intake [51], and a third did not find any improvement in child fruit and vegetable intake [49]. These prior studies were limited, however, by a small sample size, lack of a control or comparison group, or self-reported outcomes [32,50,51,52]. Larger intervention studies with more robust study designs and rigorous outcome measures are needed to fully assess the potential impact of fruit and vegetable gardens in ECE. Our study provides some justification for a future garden-based intervention in ECE in England, although we focused on perceived barriers rather than motivators to start a garden. 

We also found that space was the most common barrier reported among nurseries. Although this was not what we expected, this finding provides information that can be used to tailor a future gardening intervention. While having enough space to grow sufficient amounts of produce can be challenging, there are solutions to help overcome this challenge. In our prior study where we established gardens in ECE programs in the US [49], we used creative solutions to establish gardens in small spaces. There are numerous resources available for gardening in small or restricted areas that can apply to nurseries with insufficient space (e.g., hanging tomatoes pots) to address this barrier. 

We expected nurseries to report cost as a primary barrier to establishing gardens. In a review of gardening interventions with older children, schools reported financial challenges as a substantial impediment to growing fruits and vegetables [53]. To overcome this barrier, schools solicited donations from local businesses, held fundraising events, and applied for small grants to offset costs [53]. In a prior qualitative study in ECE, providers expressed concern about having adequate financial resources to grow fruits and vegetables sufficient to feed children [16]. Despite these prior findings, most nursery managers in our study did not express concern about costs associated with establishing fruit and vegetable gardens.

Food costs have been reported as a barrier to serving more fruits and vegetables during meals and snacks in previous studies in ECE. Growing fruit and vegetables as a way to reduce food costs may be a viable option for some ECE programs. Monsivais et al. found that increasing food expenditures in ECE settings would increase the total servings of fruits and vegetables available to children [41]. A recent intervention in US-based Head Start centers established a fruit and vegetable delivery program with local farms to provide additional low-cost fruits and vegetables to children [54]. Head Start serves mainly low-income children, and this study highlights the need to help offset costs associated with fruits and vegetables in this setting. 

However, in order to reduce food costs, gardens need to yield sufficient produce to help feed all children in care. In our prior ECE garden study, we planned for one additional serving of fruits and vegetables per week for each child [49]. Although this may not have a large impact on food costs, it could make a meaningful difference, especially over the long term if gardens are sustained. However, most of the prior gardening interventions designed for children 5 years and younger required only a modest amount of growing (e.g., seedlings in small paper cups) and did not result in full-scale gardens that would produce enough servings of fruits and vegetables for child consumption [52,55]. Thus, the majority of the existing gardening programs aimed to expose children to growing fruits and vegetables but were not designed to provide enough to help offset food costs. If a goal is to decrease costs associated with purchasing fruits and vegetables, then it is important to establish gardens that will yield sufficient produce to feed rather than just expose children.

There are some limitations to this study. First, we obtained nursery manager opinions only, although we did encourage managers to speak with teachers at the nursery prior to completing the survey. However, interest in and perceived barriers to growing fruits and vegetables are likely reflective of the nursery manager and not the entire nursery staff. Responses may have differed if we had surveyed teachers (perhaps the ones more likely to care for the gardens) rather than managers. Generalizability is also limited by the response rate, even though responses were similar across deprivation tertiles in our sample. Despite this, our results may not be generalizable to other nurseries in England. However, our response rate is nearly identical to that of a similar survey of 211 ECE programs in New Zealand (54% versus 55%) [56]. Further, we used surveys rather than qualitative interviews or focus groups because we were interested in assessing a wide range of nurseries across England and quantitatively exploring associations rather than the range of perceptions present. However, while IMD provides information on the area where the nursery is located, it does not necessarily reflect the socioeconomic status of the children in care (i.e., some children may not live close to the nursery). Therefore, the deprivation results apply to nurseries as a business located in their specific geographic area, but not necessarily to the children within the nurseries. Finally, we asked nursery managers if they were interested in growing fruits and vegetables at the nursery. However, we did not assess motivators to starting a garden. Also, the question of interest was not specific enough to assess interest in growing sufficient amounts of fruits and vegetables to help offset food costs at the nursery. 

## 5. Conclusions

As more interventions target ECE programs, gardens have the potential to yield enough produce to serve the harvested fruits and vegetables with meals and snacks. This may have a small but meaningful impact if children eat what they grow. We found that most nurseries were interested in growing fruits and vegetables. This speaks to the potential for uptake of a future wide-scale garden-based intervention in England. Findings from this cross-sectional survey of nurseries may be used to further underpin further research examining the impact of gardens on children’s fruit and vegetable intake (e.g., a large scale cluster randomized controlled trial). A productive garden has the potential to supplement the supply of produce available for meals and snacks in ECE programs. Our prior pilot intervention found that children exposed to gardens in ECE increased their intake of vegetables [49]. Children from low socioeconomic status families are particularly at risk for poorer dietary intake [4,5,6]. Growing fruits and vegetables in ECE programs in the most deprived areas of England—those that serve children from low-income families—may help offset food costs. Gardens can help ensure that the most vulnerable children are exposed to a variety of fruits and vegetables early in childhood, which could help establish healthy consumption habits later in life.

## Figures and Tables

**Table 1:** Demographic characteristics of nurseries and managers, by deprivation tertile, in England, 2012−2013.

	Total Sample (*n* = 846)	Least Deprived (*n* = 229)	Middle Deprived (*n* = 219)	Most Deprived (*n* = 398)
**Nursery Characteristics**	Number (%)			
Facility Type				
Based in workplace	312 (43)	86 (43)	82 (41)	144 (44)
Run by non-profit	180 (25)	57 (29)	50 (25)	73 (22)
Part of corporation or chain	238 (33)	57 (29)	68 (34)	113 (34)
Located in urban area	691 (82)	152 (66)	157 (72)	382 (96)
	Mean (SD)			
Years in operation	17.1 (12.3)	19.0 (12.8)	17.9 (11.6)	15.5 (12.2)
Number of children enrolled	49.9 (37.3)	46.9 (33.9)	46.4 (37.9)	53.6 (38.5)
Number of full-time staff	8.5 (7.3)	7.9 (7.4)	7.8 (7.6)	9.1 (7.0)
Number of classrooms	2.5 (1.8)	2.3 (1.6)	2.6 (1.9)	2.7 (1.8)
**Manager Characteristics**	Number (%)			
Gender, female	797 (97)	219 (97)	205 (96)	373 (96)
Education				
Less than 2-year degree	331 (42)	89 (42)	97 (47)	145 (39)
2-year degree or higher	459 (58)	122 (58)	109 (53)	228 (61)
	Mean (SD)			
Age, years	43.0 (11.1)	43.3 (10.8)	42.9 (11.6)	42.9 (11.1)
Years worked in child care field	17.2 (9.3)	16.5 (9.1)	17.1 (9.3)	17.7 (9.5)
Years worked in nursery	9.9 (7.4)	9.5 (7.3)	10.3 (7.3)	9.9 (7.4)

**Table 2:** Interest in and perceived barriers to growing fruits and vegetables.

	Total Sample (*n* = 846)	Not Interested in Growing (*n* = 154 ^a^)	Interested in Growing (*n* = 675 ^a^)	
			Already (*n* = 268)	Not yet (*n* = 407)
	Number (%)			
**Deprivation Level**				
Least deprived	229 (27)	45 (29)	74 (28)	107 (26)
Middle deprived	219 (26)	33 (21)	75 (28)	106 (26)
Most deprived	398 (47)	76 (49)	119 (44)	194 (47)
**Barriers**				
Space	281 (42)	N/A	74 (28)	207 (51)
Expertise	178 (26)	N/A	41 (15)	137 (34)
Time	106 (16)	N/A	29 (11)	77 (19)
Cost	9 (1)	N/A	1 (0)	8 (2)
Ownership/shared use	24 (4)	N/A	5 (12)	19 (5)
Seasonality/growing conditions	19 (3)	N/A	9 (3)	10 (3)
External threats	11 (2)	N/A	3 (1)	8 (2)
No barriers	165 (25)	N/A	93 (35)	72 (18)

^a^ 17 nurseries did not respond and were therefore not included as not interested or interested.

**Table 3:** Adjusted ^a^ odds ratios and 95% confidence intervals (CI) of perceived barriers to growing for nurseries interested in growing ^b^ (*n* = 407).

	Odds Ratio (95% CI)	*P*-value
**Time**		
Least deprived	Ref	Ref
Middle deprived	1.71 (0.71, 4.14)	0.23
Most deprived	1.24 (0.55, 2.83)	0.60
**Expertise**		
Least deprived	Ref	Ref
Middle deprived	1.26 (0.64, 2.49)	0.51
Most deprived	1.68 (0.91, 3.11)	0.10
**Space**		
Least deprived	Ref	Ref
Middle deprived	1.43 (0.75, 2.71)	0.28
Most deprived	2.02 (1.12, 3.66)	0.02
**Cost**		
Least deprived	Ref	Ref
Middle deprived	1.22 (0.19, 7.82)	0.84
Most deprived	0.49 (0.07, 3.75)	0.50
**External Threats**		
Least deprived	Ref	Ref
Middle deprived	0.44 (0.04, 5.01)	0.51
Most deprived	0.98 (0.17, 5.69)	0.98
**Ownership/Shared Use**		
Least deprived	Ref	Ref
Middle deprived	1.32 (0.35, 4.95)	0.68
Most deprived	0.71 (0.18, 2.81)	0.62
**Seasonality/Growing Conditions**		
Least deprived	Ref	Ref
Middle deprived	0.72 (0.13, 3.99)	0.71
Most deprived	0.22 (0.02, 2.25)	0.20
**No Barriers**		
Least deprived	Ref	Ref
Middle deprived	0.54 (0.24, 1.22)	0.14
Most deprived	0.51 (0.25, 1.06)	0.07

^a^ Adjusted for type of facility, total number of children enrolled, years in operation, and manager education. ^b^ Among nurseries interested but not yet growing fruits and vegetables.
